# What Variables Allow the Differentiation between More and Less Successful Adolescent Volleyball Players?

**DOI:** 10.5114/jhk/166107

**Published:** 2023-07-15

**Authors:** Mario Albaladejo-Saura, Raquel Vaquero-Cristóbal, Juan A. García-Roca, Francisco Esparza-Ros

**Affiliations:** 1 International Kinanthropometry Chair, Universidad Católica de Murcia (UCAM), Murcia, Spain.; 2 Faculty of Sport Sciences, Universidad Católica de Murcia (UCAM), Murcia, Spain.; 3 Centre for Olympic Studies, Universidad Católica de Murcia (UCAM), Murcia, Spain.

**Keywords:** body composition, youth, team sport, performance

## Abstract

Kinanthropometric and physical fitness variables have been habitually used for the detection of sports talent in adolescent populations. Considering these variables, players who obtained better scores than their peers in kinanthropometric and physical fitness variables have been traditionally selected for promotion in their teams, without taking into account the possible effect of the maturation process. The aim of the present study was to analyse the differences between adolescent volleyball players in terms of success assessment and the age category, as well as to identify variables that may predict success in volleyball. A total of 101 volleyball players in the U14 age category (28 boys and 73 girls; age = 13.28 ± 0.49 years) and 99 players in the U16 age category (20 boys and 79 girls; age = 15.24 ± 0.59 years) participated in the study. Significant differences were observed in biological maturation in male groups (p < 0.001–0.041), kinanthropometric variables related to bone structure and muscle mass in male groups (p < 0.001–0.048), in variables related to adipose tissue in the U16 female group (p = 0.012–0.032) and in physical fitness tests related to muscle strength and power (p < 0.001–0.049), indicating that more successful (MS) players showed a more advanced biological maturation process, higher values of kinanthropometric variables related to bone structure and muscle mass, and lower values in variables related to adipose tissue, as well as higher levels of physical fitness. The difference in biological maturation and its relationship with structural and muscular anthropometric variables in boys, and physical fitness tests related to muscle strength and power in both boys and girls, are of great importance in the selection process of sports talent in volleyball. These findings should be considered by clubs and coaches, who could be discarding players who could improve their sports performance in later stages when their maturational development is equalised.

## Introduction

The main objective of sports talent identification programmes is the early selection of players who could potentially succeed at the professional level in their sport discipline (Larkin and O'Connor, 2017). Thus, this topic has attracted the interest of the scientific community in recent years. Sports talent identification programmes have traditionally included kinanthropometric and physical performance variables (Albaladejo-Saura et al., 2021). Following these characteristics, many talent identification programmes have based their selection on size and physique, dividing athletes by age groups without considering their maturational status.

The maturation process occurs at different rates between individuals, with a reported range for the onset of maturation from 9 to 15.8 years of age (Malina and Bouchard, 1991). As a consequence of the hormonal changes caused by the maturation process in both males and females, and their influence on body shape and composition, early maturers tend to obtain higher values in kinanthropometry and fitness tests (Albaladejo-Saura et al., 2021). Given that these variables are commonly used in the detection of sports talent, together with technical-tactical skills, the different rates in maturational development could affect the selection process of sports talents. This process could prioritise the selection of individuals whose maturation process is more advanced, as they have a competitive advantage over their peers (Rubajczyk and Rokita, 2020), without considering that these characteristics could become equal as the maturation process continues. Thus, traditional models tend to exclude athletes whose maturation process is delayed (Vaeyens et al., 2008). Not surprisingly, previous research has concluded that early recruitment does not always guarantee sport success, and that it is possible that until after the age of 14, the most successful players will not stand out from their peers in terms of physical abilities (Dugdale et al., 2021c). As a consequence of the above, biological maturation has been recently included in sports talent detection research and programmes, when athletes are in the growth stage (Albaladejo-Saura et al., 2021).

Furthermore, it should be considered that kinanthropometric and physical fitness variables with the greatest influence on sport performance may depend on the specific requirements of the discipline (Carter, 1982). In this sense, volleyball is characterised by explosive actions, which makes physical fitness of great significance for elite performance (Huang et al., 2016). Due to the rules of the sport, body height, the arm span and leg length differentiate elite players (Zhao et al., 2019) from others, together with specific physical abilities such as vertical jumping, coordination and agility (Rubajczyk and Rokita, 2020), all of which are influenced by maturation (Albaladejo-Saura et al., 2021). This means that during growth, coaches who look for immediate performance may select athletes whose maturational process is more advanced, since they have competitive advantages over their teammates, as in other sports such as football or combat sports (Dugdale et al., 2021a).

Notwithstanding the above, no studies have considered this phenomenon in adolescent populations of volleyball players. Therefore, the aim of this study was to analyse the differences between adolescent volleyball players with different assessments of success in the U14 and U16 categories, as well as to identify variables that could better predict success in both age groups.

## Methods

### Sample Size

Sample size calculations were performed before the start of the study with Rstudio software (version 3.15.0, Rstudio Inc., Boston, MA, USA). The significance level was set at α = 0.05. The standard deviation (SD) was set based on the Maturity Offset from previous studies (SD = 0.26) (Arede et al., 2019). With an estimated error (d) of 0.052 years of Maturity Offset, the estimated sample needed was 99 subjects per age category. In addition, sample size calculations were performed for both boys and girls in each age and success categories, based on the Maturity Offset of studies conducted with similar populations (Maturity Offset SD = 0.87) (Albaladejo-Saura et al., 2022a). With an estimated error (d) of 0.54 years of Maturity Offset, the minimum sample needed for each group was 9 players.

### Participants

A total of 101 U14 players (28 boys and 73 girls; age = 13.28 ± 0.49 years old), and 99 U16 players (20 boys and 79 girls; age = 15.24 ± 0.59 years old) from the 1^st^ Regional Division of volleyball in Murcia, took part in the study. The reason for including a higher number of girls in both age groups was the number of teams in each league and therefore, the available sample universe, with 12 girls' teams and from 6 to 8 boys' teams for each of the selected categories. The classification of participants into two age groups was due to the age ranges established by the competent Volleyball Federation for official competitions.

Before starting the study, coaches, parents and players were informed about the measurement procedures, and an informed consent form was signed by parents or legal guardians of participants. The inclusion criteria were: a) to take part in regular volleyball training, at least two days per week; b) to participate in federated competitions; c) to be between 12 and 15 years old; d) to have played volleyball for at least two consecutive seasons at the time of measurement. Participants were excluded if they: a) suffered an injury that prevented them from completing the tests; and b) had missed more than 25% of the training sessions in the last 3 months.

### Measures

Coaches were asked to complete a questionnaire about the role and importance of each player in their team. They had to classify players as “leading team players”, “other important players”, and “players who rarely take part in the game” (Katić et al., 2006). This classification, together with the team position in the league, was used to categorize participants into the more successful (MS) or less successful (LS) groups following the methods of Katić et al. (2006).

#### Kinanthropometric and Biological Maturation Assessment

Four basic measurements (body mass, body height, sitting height and the arm span), eight skinfolds (triceps, subscapular, biceps, iliac crest, supraspinale, abdominal, thigh and calf), six girths (arm relaxed, flexed and tensed arm, waist, hips, middle thigh and calf), five breadths (biacromial, biiliocristal, humerus, femur, and bi-styloid), three lengths (acromiale-radiale, radiale-stylion and midstylion-dactylion), and a height (iliospinale) were measured following the guidelines of the International Society for the Advancement in Kinanthropometry (ISAK) (Esparza-Ros et al., 2019). All the measurements were performed by level 2 and 3 anthropometrists accredited by the ISAK. The intra- and inter-evaluator technical error of measurements (TEM) were calculated in a sub-sample. The intra-evaluator TEM was 0.06% for the basic measurements of lengths, heights and girths; and 1.12% for skinfolds; and the inter-evaluator TEM was 0.04% for the basic measurements of lengths, heights and girths; and 2.95% for skinfolds.

The following tools were used for kinanthropometric assessments: a SECA 862 scale (SECA, Germany) with accuracy of 100 g for measuring body mass; a SECA stadiometer (SECA, Germany) with accuracy of 0.1 cm for measuring standing and sitting height; an arm span meter (Smartmet, Mexico) with accuracy of 0.1 cm for measuring the arm span; a skinfold caliper (Harpenden, UK) with accuracy of 0.2 mm for measuring skinfolds; an inextensible measuring tape (Lufkin, USA) with 0.1 cm accuracy for measuring girths; a segmometer (CESCORF, Brazil) with 0.1 cm accuracy for measuring heights and lengths; an anthropometer (Realmet, Spain) and a small girth sliding caliper (Holtain, UK) with 0.1 cm accuracy for measuring bone breadths. All the measurements were taken twice. When the difference between the first and second measurements was greater than 5% for the folds, or 1% for the other measurements, a third measurement was taken. The final value used for the analysis was the mean between measurements when there were two attempts, and the median when there were three attempts.

The final values of kinanthropometric measurements were used to calculate the variables of the body mass index (BMI), fat mass (Slaughter et al., 1988), muscle mass (Poortmans et al., 2005), bone mass (Matiegka, 1921), somatotype (Carter and Heath, 1990), ∑6 skinfolds (triceps, subscapular, supraspinale, abdominal, thigh and calf), ∑8 skinfolds (triceps, subscapular, biceps, iliac crest, supraspinale, abdominal, thigh and calf), cormic index [(sitting height/height)*100], relative arm span [(arm span/height)*100], upper limb length [acromiale-radiale length + radiale-stylion length + midstylion-dactylion length], corrected girths of the arm [arm relaxed girth − (π*triceps skinfold)], thigh [middle thigh girth − (π*thigh skinfold)] and calf [calf girth − (π*calf skinfold)], the muscle-bone index [muscle mass / bone mass] and the waist-to-hip ratio (waist girth/hip girth).

The maturity offset was calculated in accordance with the procedures from Mirwald et al. (2002), using the sex-specific formula. The result was used to calculate the age at peak height velocity (APHV) for each subject using the following formula: APHV = chronological age – maturity offset result.

#### Physical Fitness Assessment

The following fitness tests were performed: a sit-and-reach test, a back scratch test, a standing long jump, and a medicine ball throw, a counter movement jump (CMJ), a 20-m sprint, and an agility test (9-3-6-3-9). The selection of the tests and their assessment were performed according to previously-described protocols (Albaladejo-Saura et al., 2022b). Two researchers with previous experience in the assessment of physical fitness tests supervised the familiarization and assessment of these tests, with the same researcher being responsible for each test during all the measurements to avoid inter-evaluator error in the assessments. Two attempts were made for each test, with a two-minute rest interval between them, and with the final value being the mean of the two trials.

The sit-and-reach test was performed with the Acuflex Tester III (Novel Products, U.S.A), the back scratch test with a millimetre ruler (GIMA, Italy), the long jump and medicine ball throw tests with a tape measure (HaeSt, Germany) with 0.1 cm accuracy, the CMJ with a force plate (MuscleLab, Norway), the sprint test (20 m) with MySprint (Apple Inc., USA) (Romero-Franco et al., 2017), and the agility test (9-3-6-3-9) with five photocells (Microgate, Italy).

#### Design

The procedures of the present study followed a cross-sectional design, in accordance with the STROBE guidelines (Vandenbroucke et al., 2014). The institutional ethics committee of the Universidad Católica San Antonio de Murcia (UCAM) reviewed and authorised the protocol designed for data collection in accordance with the Code from the World Medical Association (Code number: CE061921). The statements of the Declaration of Helsinki were followed during the entire process. The measurements were carried out in the training hall. Participants were instructed to avoid strenuous physical exercise and to ingest a light meal 3 h prior to the measurements. First, anthropometric assessments were performed, followed by physical fitness tests, with flexibility tests performed before the warm-up. Previous research has demonstrated that completing a warm-up before flexibility tests may affect performance in the selected tests (Díaz-Soler et al., 2015), thus we followed the protocol of previous research carried out in adolescents and young adults (Albaladejo-Saura et al., 2022a, 2022b; Mayorga-Vega et al., 2014; Merino-Marban et al., 2015). The warm-up was a standardized one, consisting of 10 minutes of continuous running, followed by joint mobility and familiarization with physical fitness tests. Afterwards, the long jump, medicine ball throw, counter movement jump (CMJ), 20-m sprint, and agility tests (9-3-6-3-9) were performed in that order. This order was selected according to the recommendations from the National Strength and Conditioning Association (NSCA), based on the fatigue generated by particular tests, as well as metabolic pathways required by each of them (Coburn and Malek, 2016). Coaches were asked to complete a questionnaire regarding the importance of the player in the team.

### Statistical Analysis

A descriptive analysis of the sample was performed, including the mean and standard deviation. The distribution (Kolmogorov-Smirnov test), kurtosis and asymmetry of variables were calculated. The Levene's test was used to assess the homogeneity of variables. A MANOVA test dividing the sample by sex and age categories was performed. The effect size was calculated with partial eta squared (Ƞ^2^_p_). The significance level was set a priori at *p* < 0.05. All statistical analyses were performed with SPSS v.23 software (IBM, Endicott, NY, USA).

## Results

The descriptive statistics and differences between MS and LS players are shown in Tables 1 and 2 for U14 and U16 male players, respectively; and in Tables 3 and 4 for U14 and U16 female players, respectively.

### Biological Maturation

ANOVA showed significant differences between MS and LS players in maturity offset in both U14 and U16 male groups (*p* < 0.001–0.045), with players in the MS group showing a more advanced maturation stage (Tables 1 and 2). Regarding the APHV, only U14 male players showed significant differences (*p* = 0.003), with an earlier APHV observed in the MS group (Table 1).

**Table 1:** Descriptive analysis and comparison between MS and LS U14 male players

Variable	Group		ANOVA			
	MS (n = 9)	LS (n = 14)				
	Mean ± SD	Mean ± SD	Mean Diff ± SD	*p*	Min to Max	Ƞ^2^_p_
Maturity offset (years)	0.62 ± 0.32	−0.47 ± 0.52	1.09 ± 0.25	< 0.001*	0.58 to 1.60	0.482
APHV (years)	13.02 ± 0.35	13.65 ± 0.38	−0.63 ± 0.19	0.003*	−1.03 to −0.24	0.344
Body mass (kg)	61.96 ± 10.12	55.96 ± 9.67	6.00 ± 4.93	0.237	−4.25 to 16.26	0.066
Height (cm)	175.58 ± 8.02	163.96 ± 7.12	11.62 ± 3.69	0.005*	3.95 to 19.29	0.321
Arm spam (cm)	177.46 ± 8.96	167.06 ± 8.48	10.40 ± 4.33	0.026*	1.39 to 19.41	0.215
Sitting height (cm)	90.31 ± 2.12	83.31 ± 2.76	7.00 ± 1.34	< 0.001*	4.21 to 9.79	0.565
Upper limb length (cm)	78.53 ± 3.75	74.68 ± 3.93	3.85 ± 1.97	0.064	−0.24 to 7.94	0.154
Biacromial breadth (cm)	37.64 ± 2.02	35.42 ± 1.76	2.22 ± 0.92	0.025*	0.31 to 4.12	0.218
Biiliocristal breadth (cm)	26.17 ± 1.94	25.08 ± 1.80	1.09 ± 0.92	0.250	−0.83 to 3.01	0.062
Femur breadth (cm)	10.03 ± 0.69	9.72 ± 0.46	0.31 ± 0.26	0.254	−0.24 to 0.85	0.061
Humerus breadth (cm)	7.02 ± 0.48	6.65 ± 0.29	0.37 ± 0.17	0.041*	0.02 to 0.72	0.184
Bi−styloid breadth (cm)	5.54 ± 0.40	5.14 ± 0.33	0.40 ± 0.18	0.032*	0.04 to 0.77	0.200
Corrected arm girth (cm)	22.44 ± 3.10	21.77 ± 1.98	0.67 ± 1.13	0.560	−1.68 to 3.03	0.016
Corrected thigh girth (cm)	43.01 ± 3.57	42.64 ± 3.53	0.38 ± 1.79	0.836	−3.34 to 4.09	0.002
Corrected leg girth (cm)	31.29 ± 2.23	30.43 ± 1.75	0.86 ± 0.94	0.370	−1.09 to 2.80	0.038
Endomorphy	2.23 ± 1.19	2.92 ± 1.68	−0.69 ± 0.81	0.401	−2.37 to 0.99	0.034
Mesomorphy	3.88 ± 1.72	4.71 ± 1.30	−0.83 ± 0.70	0.250	−2.29 to 0.63	0.063
Ectomorphy	4.10 ± 2.23	3.05 ± 1.60	1.05 ± 0.88	0.246	−0.78 to 2.88	0.063
∑6 Skinfolds (mm)	58.64 ± 27.62	69.90 ± 32.27	−11.26 ± 15.89	0.486	−44.31 to 21.79	0.023
∑8 Skinfolds (mm)	73.74 ± 35.43	88.64 ± 43.32	−14.90 ± 21.20	0.490	−58.99 to 29.18	0.023
Fat mass (%)	15.40 ± 6.19	18.04 ± 7.45	−2.64 ± 3.65	0.478	−10.24 to 4.95	0.024
Muscle mass (%)	37.53 ± 2.26	38.28 ± 2.50	−0.75 ± 1.24	0.550	−3.34 to 1.83	0.017
Bone mass (%)	19.49 ± 2.70	18.22 ± 2.56	1.27 ± 1.31	0.345	−1.46 to 3.99	0.043
Fat mass (kg)	9.99 ± 5.06	10.65 ± 5.88	−0.66 ± 2.90	0.822	−6.69 to 5.37	0.002
Muscle mass (kg)	23.15 ± 3.21	21.26 ± 2.80	1.89 ± 1.46	0.208	−1.14 to 4.92	0.074
Bone mass (kg)	11.94 ± 1.71	10.01 ± 0.98	1.93 ± 0.59	0.003*	0.71 to 3.15	0.341
BMI (kg/m^2^)	20.20 ± 3.89	20.82 ± 3.47	−0.62 ± 1.80	0.734	−4.36 to 3.12	0.006
Muscle−bone index	1.96 ± 0.32	2.13 ± 0.23	−0.17 ± 0.13	0.197	−0.44 to 0.10	0.078
Sit−and−reach test (cm)	4.70 ± 6.22	−2.14 ± 9.04	6.84 ± 4.33	0.129	−2.17 to 15.85	0.106
Back scratch test (cm)	0.17 ± 6.49	2.74 ± 7.56	−2.57 ± 3.73	0.498	−10.32 to 5.18	0.022
Long jump (m)	2.10 ± 0.13	1.79 ± 0.17	0.31 ± 0.08	0.001*	0.14 to 0.48	0.406
Medicine ball throw (m)	5.99 ± 1.09	5.02 ± 0.81	0.97 ± 0.44	0.039*	0.05 to 1.89	0.188
CMJ (cm)	27.85 ± 7.58	26.56 ± 3.53	1.29 ± 2.32	0.585	−3.54 to 6.11	0.014
CMJ power (W)	698.75 ± 119.83	621.49 ± 98.28	77.26 ± 51.94	0.152	−30.74 to 185.27	0.095
20 m sprint (s)	3.80 ± 0.22	3.97 ± 0.26	−0.18 ± 0.13	0.177	−0.44 to 0.09	0.085
Agility test (s)	8.90 ± 0.37	9.44 ± 0.80	−0.54 ± 0.37	0.163	−1.32 to 0.24	0.090

MS: More successful; LS: Less successful; *: Statistically significant differences.

None of the maturation-related variables showed differences in female groups.

### Kinanthropometry

Significant differences were found in the male U14 group between MS and LS players in height, the arm span, sitting height, biacromial, humerus and bi-styloid bone breadths and bone masses (*p* < 0.001–0.041) (Table 1), with MS players showing higher values. In U16 male players, significant differences were observed in the percentage of muscle mass (*p* = 0.040), where MS players obtained a higher value (Table 2).

**Table 2:** Descriptive analysis and comparison between MS and LS U16 male players.

Variable	U16 male Group		ANOVA			
	MS (n = 16)	LS (n = 9)				
	Mean ± SD	Mean ± SD	Mean Diff ± SD	*p*	Min to Max	Ƞ^2^_p_
Maturity offset (years)	1.58 ± 0.76	0.99 ± 0.56	0.59 ± 0.29	0.045*	−0.01 to 1.19	0.152
APHV (years)	13.60 ± 0.79	13.68 ± 0.35	−0.09 ± 0.28	0.763	−0.66 to 0.49	0.004
Body mass (kg)	68.58 ± 15.55	65.35 ± 12.21	3.23 ± 6.03	0.597	−9.24 to 15.71	0.012
Height (cm)	176.08 ± 8.40	171.96 ± 7.12	4.13 ± 3.33	0.227	−2.75 to 11.00	0.063
Arm spam (cm)	177.62 ± 10.03	175.69 ± 9.34	1.93 ± 4.08	0.641	−6.51 to 10.37	0.010
Sitting height (cm)	91.01 ± 4.52	88.58 ± 2.39	2.44 ± 1.63	0.149	−0.94 to 5.81	0.088
Upper limb length (cm)	79.36 ± 3.74	78.87 ± 4.03	0.50 ± 1.60	0.759	−2.82 to 3.81	0.004
Biacromial breadth (cm)	38.87 ± 3.12	37.53 ± 1.59	1.34 ± 1.12	0.243	−0.98 to 3.66	0.059
Biiliocristal breadth (cm)	26.97 ± 2.19	26.55 ± 2.06	0.43 ± 0.89	0.639	−1.43 to 2.27	0.010
Femur breadth (cm)	10.11 ± 0.56	9.69 ± 0.56	0.42 ± 0.23	0.086	−0.07 to 0.90	0.122
Humerus breadth (cm)	7.08 ± 0.38	6.82 ± 0.42	0.25 ± 0.17	0.138	−0.09 to 0.59	0.093
Bi−styloid breadth (cm)	5.48 ± 0.27	5.30 ± 0.27	0.18 ± 0.11	0.126	−0.06 to 0.42	0.099
Corrected arm girth (cm)	24.34 ± 3.28	23.42 ± 2.73	0.92 ± 1.29	0.483	−1.75 to 3.59	0.022
Corrected thigh girth (cm)	46.93 ± 4.89	44.59 ± 4.39	2.34 ± 1.97	0.247	−1.73 to 6.41	0.058
Corrected leg girth (cm)	33.37 ± 1.99	31.87 ± 2.25	1.50 ± 0.87	0.098	−0.30 to 3.30	0.115
Endomorphy	2.51 ± 1.76	3.08 ± 1.73	−0.57 ± 0.73	0.442	−2.08 to 0.94	0.026
Mesomorphy	4.75 ± 1.16	4.37 ± 1.30	0.39 ± 0.50	0.450	−0.65 to 1.43	0.025
Ectomorphy	4.05 ± 3.65	2.89 ± 1.53	1.16 ± 1.28	0.374	−1.49 to 3.82	0.034
∑6 Skinfolds (mm)	63.98 ± 42.86	69.36 ± 28.99	−5.38 ± 16.09	0.741	−38.66 to 27.90	0.005
∑8 Skinfolds (mm)	80.94 ± 54.05	88.37 ± 37.31	−7.43 ± 20.37	0.719	−49.57 to 34.71	0.006
Fat mass (%)	15.80 ± 9.21	16.92 ± 5.31	−1.12 ± 3.36	0.742	−8.07 to 5.84	0.005
Muscle mass (%)	39.74 ± 3.00	37.43 ± 1.31	2.31 ± 1.06	0.040*	0.12 to 4.50	0.171
Bone mass (%)	17.89 ± 2.46	17.03 ± 2.17	0.86 ± 0.98	0.392	−1.18 to 2.89	0.032
Fat mass (kg)	11.89 ± 10.31	11.61 ± 5.54	0.29 ± 3.73	0.940	−7.42 to 7.99	0.000
Muscle mass (kg)	27.01 ± 5.07	24.38 ± 4.09	2.63 ± 1.98	0.197	−1.46 to 6.73	0.071
Bone mass (kg)	11.95 ± 1.37	10.94 ± 1.19	1.01 ± 0.55	0.076	−0.12 to 2.14	0.130
BMI (Kg/m2)	21.96 ± 4.02	22.02 ± 3.37	−0.06 ± 1.59	0.969	−3.35 to 3.22	0.000
Muscle−bone index	2.25 ± 0.27	2.23 ± 0.26	0.03 ± 0.11	0.817	−0.20 to 0.25	0.002
Sit−and−reach test (cm)	1.53 ± 9.65	3.97 ± 4.89	−2.45 ± 3.46	0.487	−9.61 to 4.72	0.021
Back scratch test (cm)	0.45 ± 7.48	2.18 ± 7.55	−1.73 ± 3.13	0.586	−8.20 to 4.74	0.013
Long jump (m)	2.20 ± 0.32	1.88 ± 0.71	0.32 ± 0.21	0.128	−0.10 to 0.75	0.098
Medicine ball throw (m)	7.08 ± 1.52	6.69 ± 0.81	0.38 ± 0.55	0.492	−0.75 to 1.52	0.021
CMJ (cm)	32.37 ± 7.54	31.48 ± 4.55	0.89 ± 2.77	0.751	−4.84 to 6.63	0.004
CMJ power (W)	837.55 ± 180.90	790.99 ± 139.48	46.57 ± 69.86	0.512	−97.94 to 191.08	0.019
20 m sprint (s)	3.75 ± 0.28	3.70 ± 0.18	0.05 ± 0.11	0.631	−0.17 to 0.27	0.010
Agility test (s)	8.81 ± 0.59	8.62 ± 0.53	0.18 ± 0.24	0.453	−0.31 to 0.67	0.025

MS: More successful; LS: Less successful; *: Statistically significant differences.

In female groups, none of the kinanthropometric variables showed significant differences between MS and LS groups in the U14 age group (Table 3). However, in the U16 group, significant differences were observed in the endomorphic and mesomorphic components of the somatotype (*p* = 0.014–0.28), and in the ∑6 and ∑8 skinfolds (*p* = 0.012–0.032), with lower values being observed in female players classified as MS (Table 4).

**Table 3:** Descriptive analysis and comparison between MS and LS U14 female players.

Variable	Group		ANOVA			
	MS (n = 26)	LS (n = 49)				
	Mean ± SD	Mean ± SD	Mean Diff ± SD	*p*	Min to Max	Ƞ^2^_p_
Maturity offset (years)	1.26 ± 0.62	1.02 ± 0.55	0.25 ± 0.14	0.082	−0.03 to 0.52	0.041
APHV (years)	11.91 ± 0.50	11.97 ± 0.41	−0.06 ± 0.11	0.593	−0.27 to 0.16	0.004
Body mass (kg)	56.21 ± 10.67	53.39 ± 10.43	2.82 ± 2.55	0.273	−2.27 to 7.90	0.016
Height (cm)	161.32 ± 6.61	158.91 ± 5.60	2.41 ± 1.45	0.100	−0.47 to 5.30	0.037
Arm spam (cm)	162.17 ± 7.38	159.08 ± 6.66	3.09 ± 1.68	0.070	−0.25 to 6.43	0.044
Sitting height (cm)	84.12 ± 3.64	83.08 ± 3.60	1.04 ± 0.88	0.239	−0.71 to 2.79	0.019
Upper limb length (cm)	72.03 ± 3.48	71.32 ± 2.91	0.71 ± 0.76	0.350	−0.80 to 2.22	0.012
Biacromial breadth (cm)	34.92 ± 2.06	34.34 ± 1.87	0.58 ± 0.47	0.221	−0.36 to 1.52	0.020
Biiliocristal breadth (cm)	26.25 ± 2.32	25.49 ± 1.94	0.76 ± 0.50	0.135	−0.24 to 1.77	0.030
Femur breadth (cm)	9.05 ± 0.46	9.01 ± 0.56	0.04 ± 0.13	0.747	−0.22 to 0.30	0.001
Humerus breadth (cm)	6.33 ± 0.37	6.23 ± 0.37	0.10 ± 0.09	0.292	−0.08 to 0.28	0.015
Bi-styloid breadth (cm)	4.90 ± 0.26	4.92 ± 0.27	−0.02 ± 0.06	0.732	−0.15 to 0.11	0.002
Corrected arm girth (cm)	20.63 ± 2.30	20.00 ± 2.14	0.63 ± 0.53	0.241	−0.43 to 1.69	0.019
Corrected thigh girth (cm)	41.95 ± 3.99	40.37 ± 4.82	1.58 ± 1.11	0.157	−0.62 to 3.78	0.027
Corrected leg girth (cm)	28.75 ± 2.30	28.00 ± 2.78	0.75 ± 0.64	0.244	−0.52 to 2.02	0.019
Endomorphy	3.95 ± 1.24	4.07 ± 1.56	−0.12 ± 0.35	0.736	−0.83 to 0.59	0.002
Mesomorphy	4.00 ± 0.92	3.96 ± 1.30	0.05 ± 0.29	0.870	−0.53 to 0.62	0.000
Ectomorphy	2.47 ± 1.16	2.60 ± 1.60	−0.12 ± 0.35	0.728	−0.83 to 0.58	0.002
∑6 Skinfolds (mm)	87.56 ± 21.97	88.12 ± 30.88	−0.55 ± 6.83	0.936	−14.17 to 13.06	0.000
∑8 Skinfolds (mm)	110.08 ± 29.10	111.08 ± 39.84	−1.01 ± 8.86	0.910	−18.67 to 16.65	0.000
Fat mass (%)	24.71 ± 4.51	24.37 ± 6.75	0.34 ± 1.48	0.820	−2.60 to 3.28	0.001
Muscle mass (%)	31.11 ± 2.05	30.47 ± 2.89	0.63 ± 0.64	0.324	−0.64 to 1.91	0.013
Bone mass (%)	16.27 ± 1.86	16.86 ± 2.43	−0.60 ± 0.55	0.279	−1.69 to 0.49	0.016
Fat mass (kg)	14.14 ± 4.61	13.51 ± 6.07	0.64 ± 1.36	0.642	−2.08 to 3.35	0.003
Muscle mass (kg)	17.47 ± 3.40	16.22 ± 3.29	1.25 ± 0.81	0.125	−0.36 to 2.86	0.032
Bone mass (kg)	9.00 ± 1.02	8.80 ± 0.90	0.20 ± 0.23	0.382	−0.26 to 0.66	0.010
BMI (kg/m^2^)	21.49 ± 3.08	21.06 ± 3.43	0.44 ± 0.81	0.589	−1.17 to 2.04	0.004
Muscle-bone index	1.94 ± 0.27	1.84 ± 0.27	0.10 ± 0.07	0.123	−0.03 to 0.23	0.032
Sit-and-reach test (cm)	6.82 ± 7.14	4.39 ± 7.86	2.42 ± 1.85	0.194	−1.26 to 6.11	0.023
Back scratch test (cm)	4.37 ± 4.13	3.93 ± 5.16	0.44 ± 1.17	0.710	−1.90 to 2.78	0.002
Long jump (m)	1.64 ± 0.16	1.57 ± 0.22	0.08 ± 0.05	0.111	−0.02 to 0.17	0.034
Medicine ball throw (m)	5.18 ± 0.82	4.40 ± 1.02	0.78 ± 0.23	0.001*	0.31 to 1.24	0.133
CMJ (cm)	24.33 ± 3.12	22.87 ± 4.40	1.46 ± 0.97	0.139	−0.48 to 3.40	0.030
CMJ power (W)	595.85 ± 91.51	548.66 ± 103.42	47.19 ± 24.14	0.049*	−0.92 to 95.31	0.050
20 m sprint (s)	4.18 ± 0.25	4.21 ± 0.36	−0.04 ± 0.08	0.643	−0.20 to 0.12	0.003
Agility test (s)	9.27 ± 0.63	9.22 ± 1.40	0.05 ± 0.29	0.872	−0.53 to 0.63	0.000

MS: More successful; LS: Less successful; *: Statistically significant differences.

**Table 4:** Descriptive analysis and comparison between MS and LS U16 female players.

Variable	Group		ANOVA			
	MS (n = 34)	LS (n = 43)				
	Mean ± SD	Mean ± SD	Mean Diff ± SD	*p*	Min to Max	Ƞ^2^_p_
Maturity offset (years)	2.51 ± 0.49	2.52 ± 0.55	−0.01 ± 0.12	0.917	−0.25 to 0.23	0.000
APHV (years)	12.71 ± 0.40	12.73 ± 0.41	−0.03 ± 0.09	0.778	−0.21 to 0.16	0.001
Body mass (kg)	57.62 ± 8.13	59.82 ± 8.49	−2.20 ± 1.91	0.254	−6.01 to 1.61	0.017
Height (cm)	164.08 ± 5.47	163.53 ± 5.95	0.54 ± 1.32	0.681	−2.08 to 3.17	0.002
Arm spam (cm)	166.14 ± 6.19	163.94 ± 7.59	2.20 ± 1.61	0.175	−1.01 to 5.41	0.024
Sitting height (cm)	85.96 ± 2.53	85.86 ± 3.61	0.10 ± 0.73	0.887	−1.35 to 1.56	0.000
Upper limb length (cm)	74.04 ± 3.07	73.37 ± 3.24	0.67 ± 0.73	0.359	−0.78 to 2.12	0.011
Biacromial breadth (cm)	35.89 ± 1.62	35.44 ± 1.75	0.45 ± 0.39	0.253	−0.33 to 1.22	0.017
Biiliocristal breadth (cm)	26.81 ± 1.46	26.94 ± 1.47	−0.13 ± 0.34	0.704	−0.80 to 0.54	0.002
Femur breadth (cm)	9.01 ± 0.39	9.18 ± 0.54	−0.17 ± 0.11	0.123	−0.39 to 0.05	0.031
Humerus breadth (cm)	6.20 ± 0.32	6.32 ± 0.37	−0.13 ± 0.08	0.116	−0.29 to 0.03	0.033
Bi-styloid breadth (cm)	4.92 ± 0.26	4.94 ± 0.27	−0.02 ± 0.06	0.786	−0.14 to 0.11	0.001
Corrected arm girth (cm)	21.08 ± 1.71	21.20 ± 1.71	−0.12 ± 0.39	0.767	−0.90 to 0.67	0.001
Corrected thigh girth (cm)	41.89 ± 3.27	42.41 ± 3.78	−0.53 ± 0.82	0.522	−2.16 to 1.10	0.005
Corrected leg girth (cm)	29.67 ± 1.58	30.45 ± 2.76	−0.79 ± 0.53	0.142	−1.85 to 0.27	0.028
Endomorphy	3.49 ± 0.97	4.12 ± 1.17	−0.63 ± 0.25	0.014*	−1.13 to −0.13	0.078
Mesomorphy	3.66 ± 0.90	4.21 ± 1.17	−0.55 ± 0.24	0.028*	−1.03 to −0.06	0.063
Ectomorphy	2.66 ± 1.25	2.20 ± 1.13	0.46 ± 0.27	0.099	−0.09 to 1.00	0.036
∑6 Skinfolds (mm)	79.46 ± 18.93	91.69 ± 21.84	−12.23 ± 4.73	0.012*	−21.65 to −2.80	0.082
∑8 Skinfolds (mm)	98.68 ± 25.55	113.21 ± 31.45	−14.53 ± 6.66	0.032*	−27.79 to −1.28	0.060
Fat mass (%)	23.29 ± 4.54	25.40 ± 5.46	−2.12 ± 1.17	0.073	−4.44 to 0.21	0.042
Muscle mass (%)	31.71 ± 2.06	31.12 ± 1.95	0.59 ± 0.46	0.200	−0.32 to 1.51	0.022
Bone mass (%)	16.16 ± 1.77	15.70 ± 1.39	0.46 ± 0.36	0.201	−0.25 to 1.18	0.022
Fat mass (kg)	13.68 ± 4.20	15.42 ± 4.85	−1.73 ± 1.05	0.103	−3.82 to 0.36	0.035
Muscle mass (kg)	18.23 ± 2.54	18.61 ± 2.90	−0.38 ± 0.63	0.549	−1.64 to 0.88	0.005
Bone mass (kg)	9.21 ± 0.81	9.32 ± 0.99	−0.11 ± 0.21	0.599	−0.53 to 0.31	0.004
BMI (kg/m^2^)	21.37 ± 2.55	22.34 ± 2.66	−0.97 ± 0.60	0.112	−2.16 to 0.23	0.033
Muscle-bone index	1.98 ± 0.22	2.00 ± 0.20	−0.02 ± 0.05	0.747	−0.11 to 0.08	0.001
Sit-and-reach test (cm)	7.27 ± 8.81	4.85 ± 9.32	2.41 ± 2.09	0.252	−1.75 to 6.58	0.017
Back scratch test (cm)	5.52 ± 6.36	3.74 ± 5.52	1.78 ± 1.36	0.194	−0.92 to 4.47	0.022
Long jump (m)	1.75 ± 0.22	1.61 ± 0.17	0.14 ± 0.05	0.004*	0.05 to 0.23	0.108
Medicine ball throw (m)	5.47 ± 0.82	5.23 ± 1.01	0.25 ± 0.21	0.254	−0.18 to 0.67	0.017
CMJ (cm)	27.16 ± 5.00	24.65 ± 3.48	2.51 ± 0.97	0.011*	0.59 to 4.44	0.083
CMJ power (W)	647.11 ± 101.08	641.28 ± 95.71	5.84 ± 22.52	0.796	−39.02 to 50.69	0.001
20 m sprint (s)	4.03 ± 0.26	4.23 ± 0.23	−0.21 ± 0.06	< 0.001*	−0.32 to −0.10	0.159
Agility test (s)	9.18 ± 0.75	9.38 ± 0.96	−0.20 ± 0.20	0.331	−0.60 to 0.20	0.013

MS: More successful; LS: Less successful; *: Statistically significant differences.

### Physical Fitness Tests

When physical fitness tests were analysed, significant differences were found in the performance of the long jump and the medicine ball throw in the U14 male group (*p* = 0.001–0.039) (Table 1); in the medicine ball throw and CMJ power in the U14 female group (*p* = 0.001–0.049) (Table 3); and in the long jump, the CMJ and the sprint in the U16 female group (*p* < 0.001–0.011) (Table 4). In every case, MS players showed a better performance than LS ones. No differences were found in the U16 male group regarding physical fitness tests.

## Discussion

classification as MS or LS, considering both kinanthropometric and physical fitness variables. It was observed that in both sex groups, players classified as MS showed more favourable values for sports performance. In relation to biological maturation, significant differences were observed between MS and LS athletes in maturity offset in male players, but not in female players. Also, significant differences were found between MS and LS players in the APHV in the U14 male group. In every case, MS athletes showed a more advanced maturation process. The process of classifying players as MS or LS depends on both the coach's criteria and the team's performance in competitions (Katić et al., 2006). These results are in line with previous research, which highlighted that players whose maturation was more advanced were more likely to be better rated and selected for promotion (Dugdale et al., 2021a; Rubajczyk and Rokita, 2020). In addition, the method for calculating biological maturation is based on the difference between chronological age and the estimated age at which the APHV is reached (Mirwald et al., 2002), which could explain the differences when comparing the two age categories. Also, it is known that boys and girls have different age ranges around the APHV (Mirwald et al., 2002). In this sense, it has been observed that for girls, age at which the APHV occurs is usually between 11 and 14 years old, while for boys it usually occurs between 13 and 16 years old (Malina and Bouchard, 1991). Previous studies have observed that differences caused by maturation are most notable around the APHV, and have a tendency to equalize as subjects move adulthood (Dugdale et al., 2021b; Figueiredo et al., 2011; Malina and Bouchard, 1991). In the present study, girls in both age categories had passed the APHV, which could help explain the absence of differences in terms of the maturation of MS and LS players.

When kinanthropometric variables were compared, significant differences were observed in structural variables (height, the arm spam, sitting height, biacromial, humerus and bi-styloid breadths, bone mass in U14 boys) and the muscle mass percentage in U16 boys between MS and LS players in favour of the MS group. The differences found in muscle mass and bone structure variables between MS and LS players could be associated with differences in biological maturation between groups (Albaladejo-Saura et al., 2021), as MS players showed a more advanced maturation process. In regard to this finding, there were no differences between MS and LS players in the maturation-related variables in females, what could explain the absence of significant differences in muscle and bone kinanthropometric variables. The development of muscle mass appears to be linked to biological maturation, as it has been shown that the increase in muscle mass during adolescence is related to the increase in circulating testosterone during this period of time (Handelsman et al., 2018). Similarly, a marked increase in bone development is observed in the pubertal stage, influenced by growth hormone (GH), which gradually increases until adulthood (Ohlsson et al., 1998). Muscle mass has been shown to be of great importance in athletic performance due to its relationship with strength and power production (Holway and Garavaglia, 2009). Bone mass is also a key component due to its structural role in muscle development (Holway and Garavaglia, 2009). In this regard, in a sport such as volleyball, greater height and wingspan, as well as greater leg muscle mass, could be key factors influencing sports performance (Zhao et al., 2019), as these could provide a competitive advantage due to the characteristics of the game. In fact, the arm span alone could specifically allow the differentiation of elite players (Zhao et al., 2019). Regarding kinanthropometric variables, in girls, significant differences were only observed in the U16 group, in mesomorphy, endomorphy and ∑6 and ∑8 skinfolds, with MS players showing lower values. In sports involving explosive movements such as jumping, it has been observed that an excess of adipose tissue could hinder performance due to excessive weight (Cabañas and Esparza, 2009; Tanda and Knechtle, 2013). It is known that female sex hormones are closely linked to adipose tissue (Sandhu et al., 2005), and in this sense, it has been observed that the amount and distribution of adipose tissue present in adolescents is associated with circulating female sex hormones, which reach a peak concentration after the pubertal stage (Garnett et al., 2004). On the other hand, adipose tissue has been shown to be very sensitive to interventions based on physical exercise or nutrition, with most of them aiming to ensure that athletes have an adequate amount of adipose tissue for the discipline they practice (Albaladejo et al., 2019; Vaquero-Cristóbal et al., 2018). In addition, it has been observed that variables related to adipose tissue could help differentiate between elite and non-elite female athletes, with elite female athletes showing a lower adipose tissue content (Sedano et al., 2009). All this could help understand the differences found in players from the U16 group. Therefore, the results of the present study may help identify key kinanthropometric variables when attempting to identify the future sports performance of adolescent players.

When analysing the differences in performance in physical fitness tests, it was observed that MS athletes showed significantly higher values in the long jump (U14 males and U16 females), the medicine ball throw (U14 males and females), the CMJ (U16 females), CMJ power (U14 females), and the sprint (U16 females). These results are in line with previous research indicating that the ability to produce strength and power with the upper and lower limbs could be key variables in the differentiation of volleyball players of different competitive levels (Tsoukos et al., 2019a, 2019b). Physical performance in these kinds of specific tests often requires the use of muscle strength and power, and is favoured by higher values of muscle mass (Fitts et al., 1991). Nevertheless, during adolescence, the increase in power production is not always due to an increase in muscle mass, as inter- and intra-muscular coordination and neuromuscular adaptations may also be key factors (McQuilliam et al., 2020). In fact, increases in strength in the absence of gains in body mass have a greater impact on sports performance where athletes propel their own body mass (sprinting and jumping) (McQuilliam et al., 2020), such as in volleyball, what could explain why MS players showed higher values in strength-dependant tests. The improved performance in tests that require the rapid application of force could be key for characterising elite volleyball players (Rubajczyk and Rokita, 2020). Players who during adolescence showed better performance in physical fitness tests related to muscle strength and power, could be more likely be considered more successful.

The results of the present study, both in relation to anthropometric variables and physical fitness tests, found differences in favour of MS players, and are in line with the results from previous research. In this sense, a tendency has been observed of promoting players in training stages who were bigger and displayed a better physical performance, due to the fact that they were usually chronologically or biologically older than their peers (Gil et al., 2014; Kelly et al., 2021). However, this early performance does not guarantee professional success, as these differences may even out once the maturation period is over (Dugdale et al., 2021b). Thus, given the complexity of team sports, the influence of physical abilities and kinanthropometric variables may be key for the identification of sports talent, but variables such as technical ability in sports or the cognitive and mental aspects of young athletes, should also be taken into account (Dugdale et al., 2021a).

Considering all the above, for the practical implications of the present study, the results seem to indicate that for sports such as volleyball, biological maturation, a larger bone structure (U14 players) and higher values of the muscle mass percentage (U16 players), play a crucial role in the classification of players as more or less successful, for male players close to the APHV. Players whose maturational process was more advanced are more likely to be selected, what could be associated with changes in performance in physical fitness tests related to power production that occurs throughout the maturation stage. This should be taken into account by clubs and coaches, who could be discarding players who could improve their sports performance in later stages when the maturational development is equalised, as found in other sports (Dugdale et al., 2021b). For girls, a lower value in adipose-related variables and better performance in upper and lower limbs fitness tests seem to be related to their success in volleyball. In U14 and U16 female volleyball players, maturation or the related variables appear to be less important in relation to performance, as no differences were found between MS and LS players.

Among the limitations of the present study, we find its cross-sectional design, as it only allows for the establishment of relationships between the variables analysed, and the lower participation of the male population, which resulted in small groups when players were divided into different age categories and success rates. Another limitation of this study is the use of estimation equations based on regression analysis for the assessment of biological maturation, instead of wrist and hand x-rays, considered the gold standard (Malina and Bouchard, 1991). However, some aspects of the x-ray gold standard method have been identified and should be considered. It has been proven that this method is invasive, costly, and time consuming, and moreover, it exposes participants to a significant amount of radiation (Towlson et al., 2021). Because of the potential problems of using this method, some authors have proposed using alternative, less invasive methods in adolescent populations (Towlson et al., 2021). However, equations may introduce errors in the calculation of the maturity offset, established at around 0.50–0.59, limiting its use to some extent (Malina et al., 2016). Among these methods, perhaps the most often used has been the equation by Mirwald et al. (2002), as in a recent systematic review with meta-analysis, out of seven studies that were selected to assess somatic maturation through anthropometric equations, six used this equation to classify athletes from different sports (Albaladejo-Saura et al., 2021). Because of the issues identified, the findings of the present research should be taken with caution, as they may only be applicable to a specific target population.

Future research could address the differences between successful and unsuccessful players, including larger samples of both sexes, with a longitudinal design that also considers a non-estimated maturation process, including kinanthropometric variables, physical performance tests, assessment of biological maturation, as well as sport-specific skills and cognitive variables to clarify the relationships between them and the future sports performance of players in particular stages of development.

To conclude, significant differences were observed in biological maturation, kinanthropometric variables, and physical fitness, which allowed for the characterisation and differentiation of volleyball players classified as more versus less successful. More successful players showed a greater biological maturation and higher values in anthropometric variables related to bone structure and muscle mass for boys, while girls showed lower values in adipose tissue related variables, as well as better performance in tests requiring power and muscle strength for both males and females. According to the results obtained, biological maturation in boys, and its relationship with kinanthropometric and physical characteristics, remains a key factor for the classification of players as more successful, as opposed to those whose maturation process begins later; but for girls, this question remains unsolved. This should be considered when carrying out selection processes, as clubs could be discarding players who could improve their sports performance in later stages when maturational development is equalised. On the other hand, the characterisation of physical requirements of adolescent volleyball players could shed light on physical training in these stages. Therefore, due to the multifactorial nature of sports success in team sports, more research is needed to further clarify the relationships between performance and predictor variables in the formative stages of volleyball players.
